# Sensorimotor Frequency Tagging Is Enhanced by Auditory and Audiovisual but Not Visual, Inputs During a Body‐Walking Task

**DOI:** 10.1111/psyp.70225

**Published:** 2026-01-28

**Authors:** Marta Matamala‐Gomez, Adrià Vilà‐Balló, David Cucurell, Ana Tajadura‐Jiménez, Antoni Rodriguez‐Fornells

**Affiliations:** ^1^ Department of Cognition, Development and Educational Psychology Institute of Neurosciences, University of Barcelona Barcelona Spain; ^2^ Cognition and Brain Plasticity Unit, Bellvitge Biomedical Research Institute [IDIBELL] L’Hospitalet de Llobregat Barcelona Spain; ^3^ University School of Health and Sport (EUSES) University of Girona Girona Spain; ^4^ Department of Psychology University of Girona Girona Spain; ^5^ i_mBODY lab, DEI Interactive Systems Group, Department of Computer Science and Engineering Universidad Carlos III de Madrid Leganés Spain; ^6^ UCL Interaction Centre University College London London UK; ^7^ Institució Catalana de Recerca i Estudis Avançats Barcelona Spain; ^8^ Aix‐Marseille Université, Iméra Marseille France

**Keywords:** auditory stimulation, brain dynamics, EEG frequency tagging, rhythm, sensorimotor entrainment, walking movement

## Abstract

Body movements like walking can synchronize with auditory and visual inputs presented within a periodic frequency range, peaking around 2 Hz. Some evidence has shown that the spontaneous tempo of human locomotion is around 2 Hz. The EEG frequency‐tagging approach allows us to capture the coupling of beat perception with neural brain oscillations at beat frequency. This study used EEG frequency tagging to explore brain dynamics during the perception of walking‐related sensory information in the auditory (footstep sounds) and visual (point‐light figure) modalities. Sensory inputs were delivered at different rates (1, 2, and 3.6 Hz) in rhythmic or random sequences while recording EEG activity. The experiment included three conditions: (i) auditory, (ii) visual, and (iii) audiovisual, including data from 22 participants. Results showed a main effect of rhythmic sequences compared with random sequences across all frequencies in all three auditory, visual, or audiovisual conditions. Specifically, at 2 Hz, rhythmic sequences enhanced neural entrainment in the sensorimotor cortex for auditory and audiovisual conditions. This effect was absent in the visual condition alone. Notably, 2 Hz rhythmic sequences in the audiovisual condition led to coupling with temporal, sensorimotor, and occipital regions. The study suggests that sensory auditory input related to walking movement presented at 2 Hz can mediate neural entrainment with sensorimotor areas. The findings of this study can have an impact on the spontaneous rhythmic integration of body movements using sensory inputs for walking rehabilitation.

## Abstract 2

This study explores how sensory inputs influence walking, a rhythmic movement requiring integration of various signals. Our results show increased synchronization in the sensorimotor cortex during rhythmic auditory and audiovisual conditions at 2 Hz. These findings highlight the complementary roles of auditory and visual stimuli in enhancing sensorimotor integration, with potential applications in motor learning techniques, particularly for improving rehabilitation and movement performance through rhythmic sensory cues.

## 1 Introduction

Body movements require tight synchronization between sensory inputs and motor processes (Nozaradan et al. 2015). Such synchronization has been best observed when movements are aligned with music (Janata et al. 2012; Rodriguez‐Fornells et al. 2012). Additionally, the brain can coordinate our own actions with the timing of others' actions (Sebanz et al. 2006). This coordination between bodies and minds is commonly known as joint action (Sebanz et al. 2006). A crucial aspect for synchronizing body movements with sensory inputs is the periodic nature of the sensory input, yielding optimal predictability of upcoming events in a given sequence (Phillips‐Silver and Keller 2012). This periodicity enables accurate and precise coordination of body movements with rhythmic events over time (Nozaradan 2014). Walking movement is one of the most rhythmic movements, involving multiple interactions between different sensory signals (Thaut et al. 1996). It requires effective coordination between the central and peripheral nervous systems and the musculoskeletal system, enabling both spatial (inter‐limb coordination) and temporal (rhythmicity—constancy in step repetitions) synchronization of limb movements (Clark 2015). Some evidence has shown that the spontaneous tempo of human locomotion is around 2 Hz. In detail, laboratory studies of overground human walking (Murray et al. 1964) have shown that on average preferred cadence is close to 120 steps per minute, equivalent to a step frequency of 2 Hz, whereas on a treadmill step frequency varies considerably with belt speed from 1.5 Hz for slow walking (0.6 m/s) to 2.4 Hz for fast walking (2.2 m/s) (Hirasaki et al. 1999). Rhythmic body movements, such as walking, selectively shape the neural representation of rhythm through implicit audio‐motor modulation (Chemin et al. 2014). Indeed, it has been shown that body and walking movements can be synchronized using both auditory (Nozaradan et al. 2015) or visual (Cracco et al. 2022) inputs presented within a periodic frequency range, peaking around 2 Hz.

Regarding auditory inputs, body movements can be synchronized with auditory rhythms across various contexts (Burger et al. 2014; Van Dyck et al. 2015). Importantly, for effective perception of the rhythmic sensorimotor coupling, auditory rhythms should be provided within a specific frequency range, from 0.5 to 5 Hz, with an optimal peak at 2 Hz, to induce proper entrainment responses (London 2012). This makes brain rhythms potentially ideal instruments for sensory input selection; if the high‐excitability phase of oscillations can be coupled to coincide with the rhythms of the task‐relevant sensory input, this input will receive optimal brain processing (Schroeder and Lakatos 2009). According to this, other studies have demonstrated that it is possible to synchronize sensory inputs and motor processes by using visual inputs related to walking movements (Cracco et al. 2020, 2022). In detail, brain responses in the occipital cortex coupled with the observation of a ‘point‐light walker’ moving at a periodic pace of 2.4 Hz have been observed using an electroencephalogram (EEG) frequency‐tagging approach (Cracco et al. 2022). This approach involves presenting stimuli and analyzing neural responses at the frequency rate of the stimuli presentation (Sieving et al. 1998). The response is evident as a peak of power at exactly the stimulus presentation rate (frequency) and/or its higher harmonics (Regan and Regan 1988).

**FIGURE 1:**
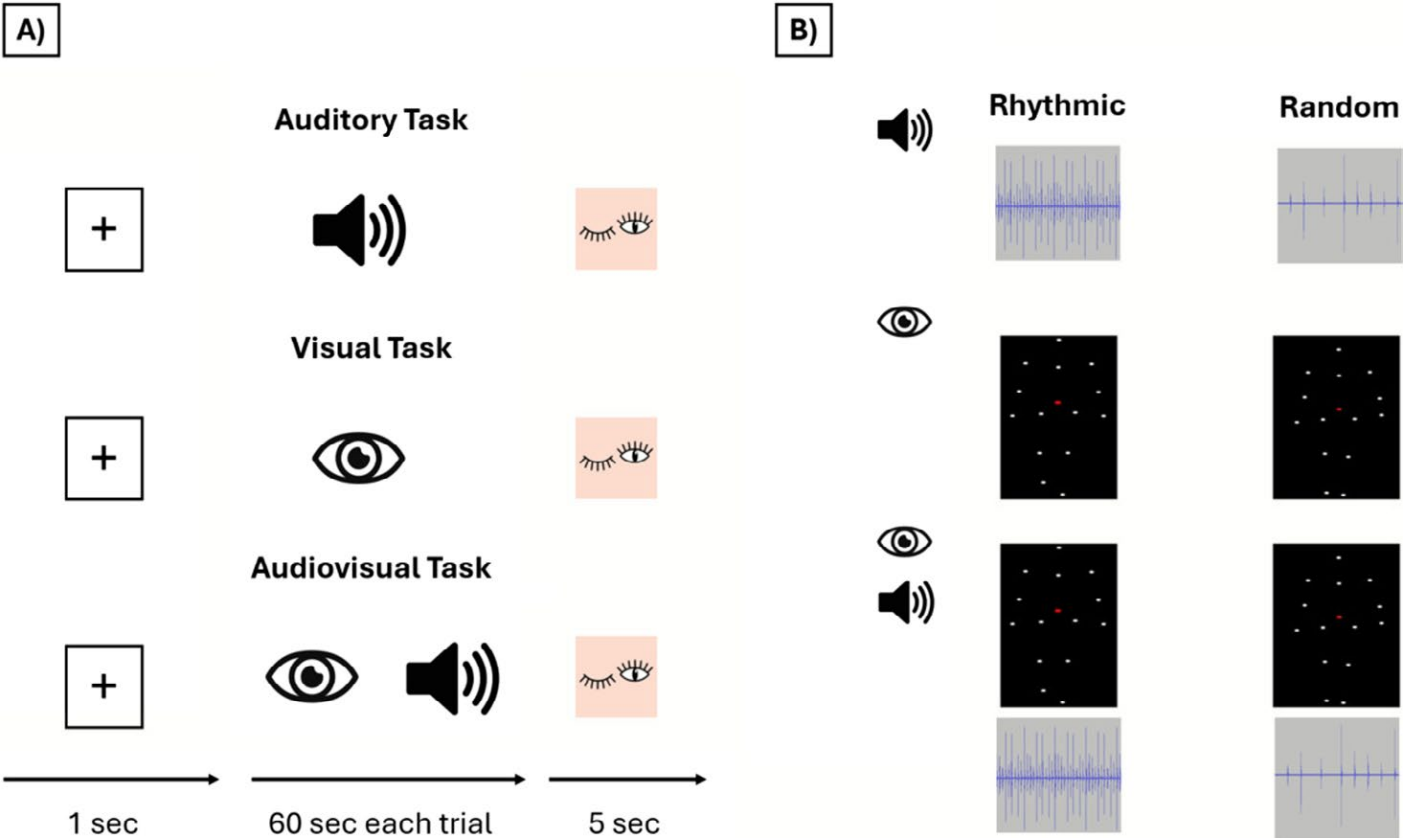
Experimental timeline and experimental conditions. (A) Summary of each trial type, experimental timeline (fixation cross, stimuli, blinking), and the experimental structure for each condition. (B) Experimental stimuli used in each condition (Auditory, Visual and Audiovisual) and its types—rhythmic and random control frequencies. Each sensory input was presented at different frequencies (1, 2, and 3.6 Hz). Sensory inputs can be observed and listened here: https://osf.io/8sa9t/files/osfstorage.

Other investigations observed that, related to walking movement integration, bimodal induction through an auditory beat and a synchronously bouncing visual stimuli through a point‐light figure induces higher beat accents and improves the reproduction of auditory rhythm in a behavioral task (Su 2014). Specifically, the visual gestalt of a walker has been shown to play a critical role in encoding the natural temporal relationship to the heard footstep sounds (Saygin et al. 2008; Troje and Westhoff 2006). However, other studies have observed that visual cues (point‐light figures) do not enhance rhythmic audio‐motor coupling (Fiveash et al. 2022). Regarding auditory stimuli, previous studies have shown that adding auditory information enhances perception of task‐relevant information and improves sensory‐motor coordination only when auditory information is congruent with visual information (a dot moving horizontally on the screen) (Rosati et al. 2012). Moreover, previous research has also shown that rhythmic visual and auditory stimulation promotes sensorimotor entrainment, with an optimal frequency around 2 Hz—which corresponds to the natural cadence of human walking. However, studies examining passive viewing and listening have reported audiovisual integration at higher frequencies (approximately 10.3 Hz for visual and 5.0 Hz for auditory stimuli). To our knowledge, no study has yet investigated the optimal frequency for integrating congruent audiovisual stimuli depicting human walking movements, nor how such integration synchronizes with activity in the sensorimotor cortex. We hypothesized that presenting audiovisual stimuli related to human walking at 2 Hz would elicit stronger frequency tagging in the sensorimotor cortex, providing insights that may inform multimodal motor rehabilitation strategies for individuals with gait disorders. It is known that frequency tagging allows us to investigate the neural integration of two different sensory inputs coming from different modalities, as reflected in increased amplitude at the intermodulation frequency components, corresponding to the addition or subtraction of the two fundamental tagging frequencies and their harmonics (Gordon et al. 2019). However, while neural responses at the intermodulation frequencies have been shown within sensory modalities (e.g., between two sensory inputs of the same modality) in other studies, evidence showing neural responses at the intermodulation frequencies across sensory modalities is still lacking. In this regard, a study from (Giani et al. 2012) compared passive perception under uni‐ and bimodal sensory conditions and found responses at the intermodulation frequencies only in the unimodal visual condition but not in the bimodal audiovisual condition. Moreover, recent studies used frequency tagging to observe neural integration for unimodal sensory conditions (e.g., visual) displayed in rhythmic or non‐rhythmic sequences (Cracco et al. 2022). The present study aims to investigate the perception of rhythmic sensorimotor entrainment when coupling auditory and visual inputs related to walking movement using an EEG frequency‐tagging approach. To that aim, auditory (footstep sounds), visual (walking point‐light figure), and audiovisual (footstep sounds + point‐light figure) inputs related to walking movement were presented at different frequency rates (1, 2, and 3.6 Hz) in rhythmic or random sequences (Figure 1).

## 2 Materials and Methods

### 2.1 Participants

Twenty‐two participants (mean age: 28.23 ± 9.46 years) were recruited from the student population at the Campus Bellvitge of the University of Barcelona (Barcelona, Spain). All participants provided informed written consent and received 30 euros for their participation, in accordance with the ethics committee of the Bellvitge Biomedical Research Institute (reference number PR288/22) and was carried out according to the Declaration of Helsinki (World Medical Association 2013). Stimuli were presented using Prime 3.0 software (Schneider et al. 2012).

### 2.2 Experimental Paradigm

This study used a mixed models design and was divided into three blocks, each containing a different task: (i) auditory task, (ii) visual task, and (iii) audiovisual task. The visual task consisted of presenting videos of a point‐light figure walking at a specific frequency rate; the auditory task consisted of presenting footstep sound stimuli at a specific frequency rate; and the audiovisual block consisted of presenting both the point‐light figure walking video and the footsteps sounds synchronized at the same frequency rate. Brain oscillatory activity was measured while manipulating two variables: the rhythm of the walking movement (rhythmic vs. random) and the frequency rate of the input presentation, using three different frequencies (only for rhythmic conditions): slow (1 Hz); normal (2 Hz); and fast (3.6 Hz). The selection of the frequencies was chosen with the aim of demonstrating the impact of 2 Hz frequency of auditory and visual inputs related to walking body movement in the sensorimotor cortices. To that, we chose one slower frequency (1 Hz) and another faster frequency (3.6 Hz) to observe how 2 Hz presentation frequency differs compared with one slower or faster frequency that were already used in previous studies (Colling et al. 2017; Nozaradan 2014; Nozaradan et al. 2015). In each block, brain responses were coupled to that frequency rate of the stimuli presentation in a rhythmic or random sequence. Each experimental block was composed of four different conditions: three rhythmic sequences (1, 2, and 3.6 Hz) and one random sequence. Each rhythmic sequence was repeated four times, then there were an overall of 12 rhythmic sequences presented in a pseudo‐random order. Likewise, the random sequence was repeated 12 times in a pseudo‐random order.

A fixation cross was shown for 1 s before the presentation of each condition. Participants were instructed not to blink during the stimuli presentation. A blink image was shown for 5 s after each condition to allow participants to blink. Each block involved a total of 24 presentations of the conditions. Each visual, auditory, and audiovisual stimulus was presented for 60 s, and each block lasted approximately 35 min (Figure 1). There was a 10–15‐min break between blocks. The order of block presentation was counterbalanced among participants.

### 2.3 Stimuli

For the visual stimuli, point‐light figures were created using the online BMLStimuli tool (Troje 2002; https://www.biomotionlab.ca/Experiments/BMLstimuli/index.html) and were shown in white against a black background. The GIF file output of the point‐light walking figure at each specific frequency rate (1, 2, and 3.6 Hz) was obtained using the speed option on the BMLStimuli tool. The walking frequency of the downloaded GIF files was double‐checked using custom‐made MATLAB scripts. A 60‐s walking video was created using Adobe Premiere Pro video editor (Adobe Systems Incorporated) by looping the five cycles gait GIF file at the selected speed downloaded from the BMLStimuli tool. For the visual random sequences' different frames of the rhythmic walking movement were cut and mixed randomly to create a 60‐s video showing a random walking movement. The 60‐s auditory stimuli consisted of pre‐recorded footstep sounds used in previous studies (Gomez‐Andres et al. 2020; Tajadura‐Jiménez et al. 2015). The footstep sound frequencies were adjusted to each specific frequency (1, 2, and 3.6 Hz) in rhythmic sequences and in the random sequences the footsteps sounds were split and mixed randomly throughout the 60‐s auditory stimuli. The auditory stimuli have been created using Audacity software (Audacity Team, Version 3.4). For the audiovisual stimuli, the visual and auditory components were synchronized at the specific frequency rates (1, 2, and 3.6 Hz) using Adobe Premiere Pro. In the random sequences the same random footsteps sound stimuli and the same random walking movement sequences used in the audio and visual blocks, were mixed without following any rhythmic pattern using Adobe Premiere Pro (see detail of the experimental stimuli here: https://osf.io/8sa9t/).

### 2.4 Experimental Procedure

Subjects were tested individually in a soundproof room. The electrophysiological session lasted 3 h. During this session, subjects were seated comfortably in a chair and were exposed to the different experimental blocks presented in a counterbalanced order while EEG was recorded. Before each block, the researcher provided detailed instructions for each task. There was a 10‐min break between each experimental block.

In the visual block, participants were instructed to observe the walking point‐light figure presented in either rhythmic sequences presented at the different frequency rates (1, 2, and 3.6 Hz) or random sequences. In the auditory task, participants listened through headphones to footstep sounds presented in rhythmic or random sequences, presented at the different frequency rates (1, 2, and 3.6 Hz). In the audiovisual task, participants were instructed to both observe and listen to the walking point‐light figures synchronized with the footstep sounds, presented in rhythmic or random sequences, presented at the different frequency rates (1, 2, and 3.6 Hz).

Each experimental block included an attentional task to ensure that participants focused their attention on the screen. In the visual and the audiovisual blocks, the central dot of the walker was colored red and served as a fixation cross. The task consisted of counting how many times the red dot turned white during the 60‐s video and reporting the count, after the presentation of each condition, through a speaker placed in front of them. In the auditory block, the attentional task consisted in detecting pitch changes (high (+0.5) or low (−0.5)) in the footstep sounds and reporting the number of pitch changes, after the presentation of each condition, through the speaker.

### 2.5 EEG Recording, Preprocessing and Processing

The EEG signal was recorded at a sampling rate of 500 Hz using two BrainAmp Standard amplifiers with 32 channels (Brain Products GmbH 2023). The electroencephalogram was recorded using a standard set‐up with 64 Ag–AgCl electrodes placed on the scalp according to the International 10/10 system (EasyCap). EEG was online filtered using a high‐pass filter of 0.016 Hz and a notch filter of 50 Hz. The reference electrode was placed 1 cm from the outer canthus of the right eye. Ocular movements were monitored with an electrode placed 1 cm below the right eye. Separate recordings were obtained for each participant, experimental block (visual, auditory, and audiovisual), rhythmic sequences at different frequency presentation (1, 2, and 3.6 Hz), and random sequences. Artifacts were corrected offline using independent components analysis (ICA) using the EEGLAB toolbox in MATLAB (MathWorks 2023). For the spectral power analyses, a current‐source density transformation was applied to the data. The data were then epoched into 20‐s time‐windows, locked at the beginning of each sequence, and baseline‐corrected using the mean activity of the whole sequence. The epochs compute the spectral power analysis, but the cycles corresponding to “standard epochs” inside the total activity are of 240 s (Cracco et al. 2022; Nozaradan et al. 2015). Spectral power was computed using the Fieldtrip toolbox in MATLAB, employing the “mtmfft” method [multi‐taper method (MTM) with a discrete prolate spheroidal sequence (DPSS) taper and a fast Fourier transform (FFT)] and using a Hanning window as the taper. Then, spectral power was normalized by dividing by the mean power across frequencies, and baseline‐corrected by first subtracting and then dividing by the mean of the adjacent bins (two on each side, spaced two bins from the target one). This baseline selection followed the approach used by Cracco et al. (2022). Specifically, since the frequency resolution is 0.05 Hz, the bins immediately adjacent to 2 Hz (i.e., 1.95 and 2.05 Hz) were not included in the baseline correction. Instead, the baseline was computed using bins from −0.10 to −0.55 Hz (1.45, 1.50, 1.55, 1.60, 1.65, 1.70, 1.75, 1.80, 1.85, and 1.90 Hz) on the lower side and from +0.10 to +0.55 Hz (2.10, 2.15, 2.20, 2.25, 2.30, 2.35, 2.40, 2.45, 2.50, and 2.55 Hz) on the upper side. The final baseline correction was performed by first subtracting the mean of these selected bins from the spectral power at 2.00 Hz and then dividing by the same mean, ensuring consistency in normalization across frequencies. Transformations of the EEG activity using current‐source density (CSD) have been employed to investigate novelty processing in healthy individuals. This method, often referred to as Laplacian transformed EEG (LT‐EEG), is widely regarded as a reference‐free technique that enhances ERP topographies in a manner that is meaningful from a physiological perspective (Amengual et al. 2014; Kayser et al. 2006).

### 2.6 Statistical Analysis

Data from 5 male and 17 women, totaling 22 participants, were analyzed. To investigate the impact of each Condition and Frequency on the effect of Rhythm in each Region of Interest (ROI), a general linear mixed‐effects model (GLMM) was fitted using the “glmer” function from the “lme4” package in R to the data of each frequency (1, 2, and 3.6 Hz). Specifically, the lme4 package (Bates et al. 2015) for the R programming language provides functions to fit and analyze linear, generalized linear, and nonlinear mixed‐effects models. Within this package, the “glmer” function is specifically designed to fit GLMMs, enabling researchers to incorporate both fixed effects (e.g., experimental conditions) and random effects (e.g., participant variability) into their models. The package also facilitates the modeling of non‐normal response distributions through the flexible specification of distribution families and link functions. In our analysis, the full models included Rhythm (with two levels: rhythmic, random), condition (with three levels: auditory, visual, audiovisual), and ROI (with three levels: sensorimotor, temporal, occipital) as within‐subject factors, along with their interactions. Participant was included as a random effect to account for individual differences. For the analyses, the three different ROIs included the following electrodes: Temporal (right: FT9, FT7, T7, TP7; left: FT10, FT8, T8, TP8), Sensorimotor (right: F5, F3, F1, FC5, FC3, FC1, C5, C3, C1; left: F2, F4, F6, FC2, FC4, FC6, C2, C4, C6), and Occipital (PO3, POz, PO4, O1, Oz, O2). Electrode selection followed a procedure commonly used in EEG research, which involved averaging the topographies across participants to identify peaks and the surrounding clusters—an approach known as the collapsed localizer method (Luck and Gaspelin 2017). To model the response variable Power—which is continuous and positively skewed—we used a Gamma distribution with an inverse link function. Model estimation was carried out using the “bobyqa” optimizer (Bound Optimization By Quadratic Approximation), a derivative‐free algorithm that iteratively adjusts model parameters to minimize the difference between predicted and observed values. This optimizer is particularly well‐suited to complex models with constraints, such as those commonly encountered in mixed‐effects modeling. We set the maximum number of function evaluations to 100,000 to ensure convergence. The inverse link function with the Gamma family was used, which is appropriate for our response variable Power that is continuous and positively skewed. The optimizer used was “bobyqa” with a maximum function evaluation of 100,000.

To test the significance of our fixed effects, we conducted a likelihood ratio test using Type II sums of squares. This was implemented using the Anova function from the car package (Fox et al. 2023). The car package provides tools for regression diagnostics and hypothesis testing in linear, generalized linear, and mixed‐effects models. Specifically, the Anova function computes Type II or Type III analysis‐of‐variance tables for a variety of model objects, including mixed‐effects models fitted with the lme4 package. It allows for flexible specification of test types and is widely used to evaluate the contribution of individual predictors and their interactions. The results of these tests are reported in the Results section of the paper.

In order to refine the models, a backward elimination procedure was applied. This is a stepwise model selection method that begins with the full model and iteratively removes non‐significant predictors or interactions based on a predefined threshold (in our case, *p* < 0.05) (Berk 1980). This methodological approach helps simplify the model while ensuring the retention of statistically significant effects. The residuals of the final models were checked to ensure they conformed to a normal distribution.

When necessary, we performed post hoc comparisons using the emmeans package in R (Lenth 2020), which provides tools for estimating and comparing marginal means derived from fitted models. Specifically, we used the emmeans function to compute estimated marginal means (also known as least‐squares means) for combinations of factors, adjusted for other variables in the model via a reference grid. Given that our model employed a Gamma distribution with an inverse link function, the estimated marginal means were initially expressed on the scale of the linear predictor. To enhance interpretability, these means were transformed back to the response scale using the regrid function, which re‐expresses marginal means on the original outcome scale when the model uses a non‐identity link function—such as log, logit, or probit—commonly used in generalized linear models. Subsequently, we conducted pairwise comparisons of the re‐scaled marginal means using the pairs' function, which tests differences between factor levels while controlling multiple comparisons. The *z*‐ratio and FDR‐corrected *p*‐values are also reported (see open‐source dataset: https://osf.io/8sa9t/). Effect sizes were calculated using “emmeans” and “effectsize” packages (Ben‐Shachar et al. 2020). The emmeans package was used to extract estimated marginal means and contrasts from the fitted models, while the effectsize package provided standardized metrics—such as Cohen's *d*, *η* ^2^, and *r*—to quantify the magnitude of these effects. Although effect size does not directly compare estimated marginal means, it complements emmeans by enabling the computation of interpretable effect sizes from model‐derived contrasts. To ensure clarity in the interpretation of the effects across the different levels of the factors, Cohen's *d* values for the main effects and interactions of the models will be reported in absolute terms.

## 3 Results

To evaluate the effect of rhythmic stimulation over the different conditions at each frequency, in each ROI, we performed GLMMs separately for each frequency. Then, we employed a backward method to eliminate non‐significant interactions or predictors. After this method, the full model was maintained for the 2 and 3.6 Hz frequencies, but not for the 1 Hz, where the triple interaction (Rhythm × Condition × ROI) was removed because it was not statistically significant (*p* > 0.05). Removing this interaction slightly affected the *p*‐values of the remaining factors and two‐way interactions, as expected in a backward elimination procedure; however, the models remain equivalent in structure and interpretation and can be compared with the 2 and 3.6 Hz frequency models. The reduced model for 1 Hz simply reflects that the triple interaction was not significant, while all other predictors were retained. The full details of the results from the three models are included in Table 1. In the Results section, we will focus on the effects of Rhythm on power amplitude for each condition and specific ROI.

**TABLE 1:** Chi‐square values and *p*‐values (Pr(>*X* ^2^)) obtained from the final model for each frequency (1, 2, and 3.6 Hz).

	Frequencies								
	1 Hz			2 Hz			3.6 Hz		
	*X* ^2^	*p*	Cohen's *d*	*X* ^2^	*p*	Cohen's *d*	*X* ^2^	*p*	Cohen's *d*
Rhythm (df = 1)	7.120	**0.008**	0.145	295.875	**< 2.2e‐16**	2.207	728.475	**< 2.2e‐16**	3.787
Condition (df = 2)	10.380	**0.006**	0.099	67.695	**1.996e‐15**	0.950	114.503	**< 2.2e‐16**	0.831
ROI (df = 2)	1.085	0.581	0.078	4.641	0.098	0.113	370.846	**< 2.2e‐16**	0.717
Rhythm × Condition (df = 2)	14.845	**5.977e‐04**	0.050	35.469	**1.986e‐08**	0.437	43.013	**4.571e‐10**	0.348
Rhythm × ROI (df = 2)	16.162	**3.094e‐04**	0.092	0.405	0.817	0.349	65.349	**6.452e‐15**	0.261
Condition × ROI (df = 4)	39.841	**4.668e‐08**	0.034	125.223	**< 2.2e‐16**	0.362	137.653	**< 2.2e‐16**	0.852
Rhythm × Condition × ROI (df = 4)	—	—	—	37.206	**1.633e‐07**	0.168	72.897	**5.548e‐15**	1.823

*Note:* This table includes the main effects (Rhythm, Condition, and ROI) and their interactions. Absolute values of Cohen's *d* are reported. Degrees of freedom (df) are indicated in parentheses. Significant *p*‐values are highlighted in bold.

### 3.1 1 Hz Frequency

**FIGURE 2:**
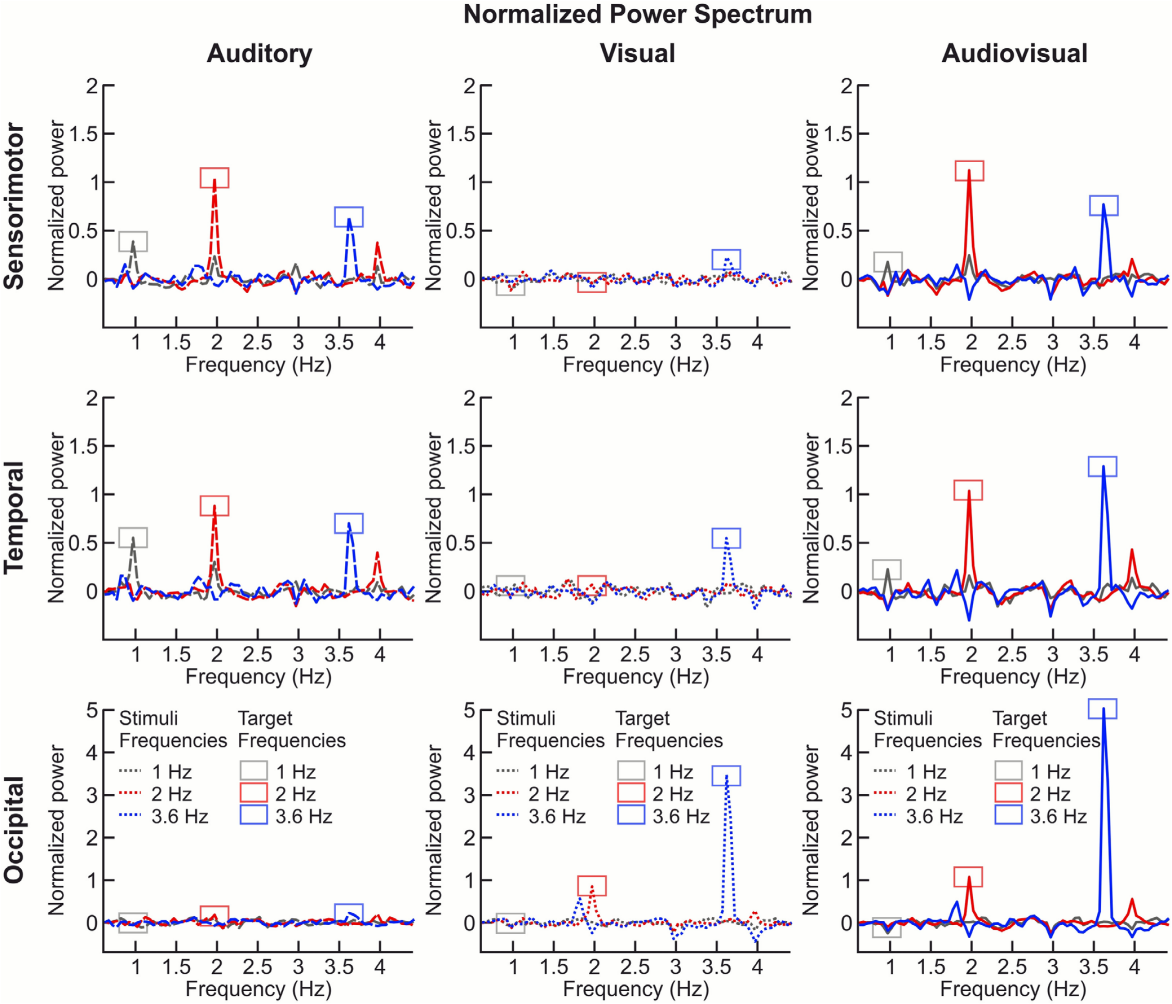
The difference in the normalized power spectrum between Rhythmic and Random sequences is depicted separately for sensorimotor (top), temporal (middle), and occipital (bottom) regions of interest (ROIs), and for auditory (left, dashed lines), visual (middle, dotted lines), and audiovisual (right, solid lines) conditions. Stimulation frequencies are represented as follows: Gray for 1 Hz, red for 2 Hz, and blue for 3.6 Hz. Rectangles indicate the target peak frequencies that will be included in Figure 3 (gray for 1 Hz, red for 2 Hz, and blue for 3.6 Hz). See Figures S1 and S2 for separate plots for Rhythmic and Random sequences.

**FIGURE 3:**
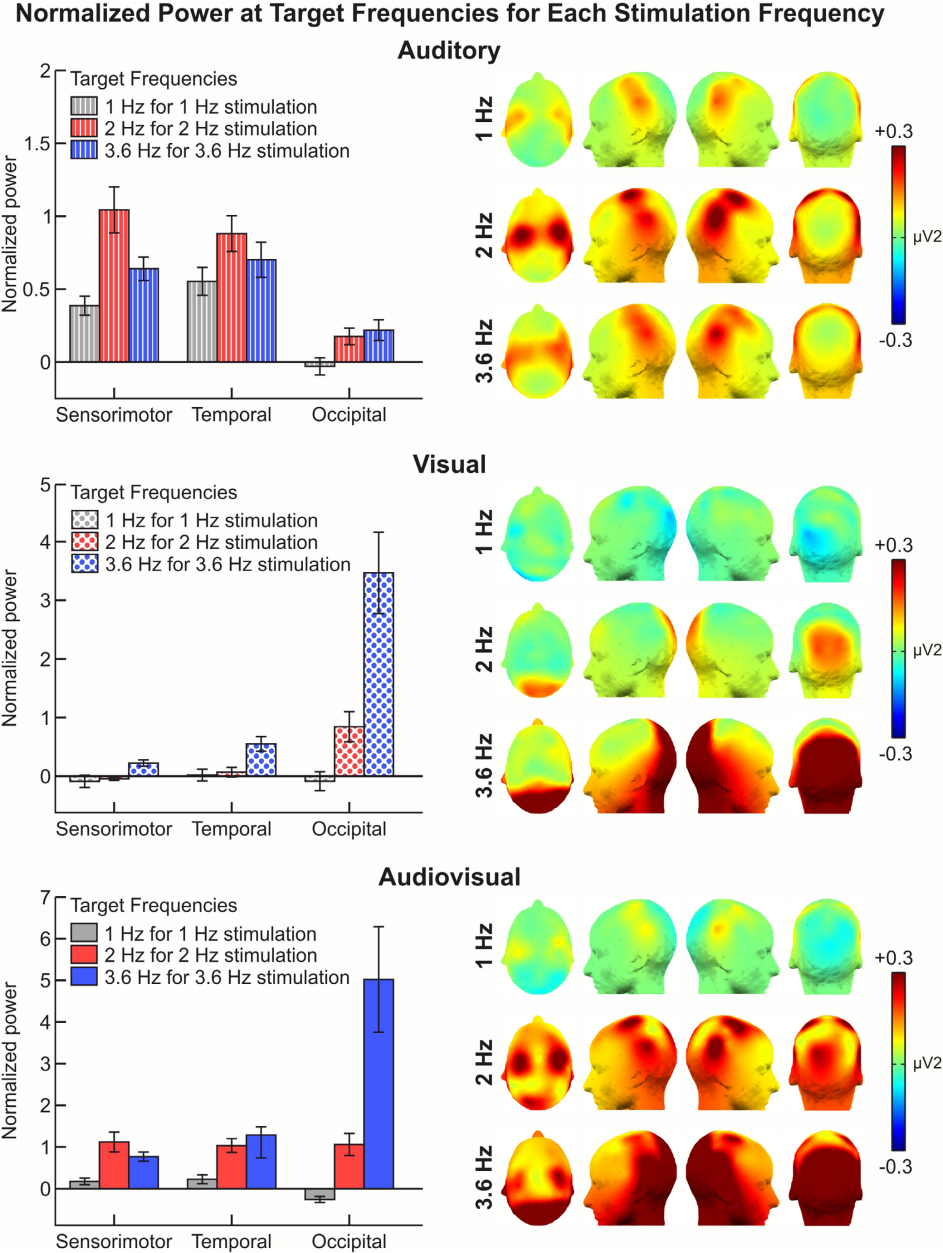
The difference in the normalized power spectrum for each target frequency (1, 2, or 3.6 Hz) at each corresponding stimulation frequency (1, 2, or 3.6 Hz) is illustrated, comparing rhythmic and random sequences. On the left, bar plots for sensorimotor, temporal, and occipital regions of interest (ROIs), as well as for auditory (top, represented with dashed bars), visual (middle, represented with dotted bars), and audiovisual (bottom, represented with solid bars) conditions, are shown. The spectral power at 1 Hz is displayed only for the stimulation frequency of 1 Hz (colored gray), at 2 Hz only for the stimulation frequency of 2 Hz (colored red), and at 3.6 Hz only for the stimulation frequency of 3.6 Hz (colored blue). Error bars represent the standard errors of the mean (SEMs). See Figure S3 for separate plots of rhythmic and random sequences.

At 1 Hz, we observed a clear peak in the sensorimotor and temporal ROIs (see Figure 2) in the auditory and audiovisual conditions. Accordingly, the average power spectrum between rhythmic and random rhythms was higher in the sensorimotor and temporal ROIs (see Figure 3) in the auditory and audiovisual conditions. No activation was shown in the Occipital ROI throughout these conditions (see Figures 2 and 3). Specifically, the statistical analyses showed a significant main effect of Rhythm, with mean Power at 1 Hz being higher for Rhythmic compared with Random sequences. Notably, there was a significant interaction between Rhythm and Condition. Post hoc pairwise contrasts comparing the effect of Rhythm at each condition revealed that the larger power for Rhythmic compared with Random was significant only in the auditory condition (*z* = 4.403, *p* = 0.003, *d* = 1.119). However, no significant effect of Rhythm was observed for the visual (*z* = −0.369, *p* = 0.712, *d* = −0.096) and audiovisual (*z* = 0.510, *p* = 0.712, *d* = 0.130) conditions. Moreover, there was a significant interaction between Rhythm and ROI, which suggested that the effect of Rhythm differed among ROIs. Post hoc analyses revealed that in both the sensorimotor (*z* = 2.496, *p* = 0.026, *d* = 0.651) and the temporal (*z* = 3.904, *p* = 0.003, *d* = 1.039) ROIs, the power was significantly higher in Rhythmic sequences as compared with Random sequences. This effect was not observed in the Occipital ROI (*z* = −1.494, *p* = 0.203, *d* = −0.365).

### 3.2 2 Hz Frequency

At 2 Hz, we observed a peak in the sensorimotor and temporal ROIs in the auditory condition, and in the occipital ROI in the visual condition. Notably, the peak was shown in all three ROIs (sensorimotor, temporal, and occipital) in the audiovisual condition (Figure 2). Accordingly, the average power spectrum between rhythmic and random sequences was higher in the sensorimotor and temporal ROIs in the auditory condition, in the occipital ROI with visual condition, and in all three ROIs (sensorimotor, temporal, and occipital) in the audiovisual condition (see Figure 3). Specifically, the statistical analyses showed a significant main effect of Rhythm, showing that the mean power at 2 Hz was higher for Rhythmic compared with Random sequences. The interactions between Rhythm and Condition, and between Rhythm and ROI were also significant. Importantly, there was a significant interaction between the three factors (Rhythm, Condition, and ROI). Post hoc pairwise analyses comparing the power between Rhythmic and Random conditions showed that in the auditory condition the effect of Rhythm was significant in the sensorimotor (*z* = 7.37, *p* = 0.002, *d* = 4.120) and the temporal (*z* = 6.816, *p* = 0.002, *d* = 3.500) ROIs. In the visual condition, the effect of Rhythm was only significant in the occipital ROI (*z* = 5.863, *p* = 0.002, *d* = 3.296). However, in audiovisual condition the effect of Rhythm was significant in all ROIs; sensorimotor (*z* = 7.108, *p* = 0.002, *d* = 4.370), temporal (*z* = 6.716, *p* = 0.002, *d* = 4.030), and occipital (*z* = 6.667, *p* = 0.002, *d* = 4.120).

### 3.3 3.6 Hz Frequency

At 3.6 Hz, we observed a peak in the sensorimotor and temporal ROIs in the auditory condition. Notably, in the visual condition, the results showed an activation peak in all three ROIs (sensorimotor, temporal, and occipital), with a larger peak in the occipital ROI. Further, in the audiovisual condition, the results showed even higher activation peaks in all three ROIs compared with the visual condition (see Figure 2). Accordingly, the average power spectrum between rhythmic and random sequences was higher in the sensorimotor and temporal ROIs in the auditory condition, in the occipital ROI in the visual condition, and in all three ROIs (sensorimotor, temporal, and occipital) in the audiovisual conditions (Figure 3). Specifically, the statistical analyses showed a significant main effect of Rhythm, with mean power at 3.6 Hz being higher for Rhythmic compared with Random sequences. Both the interaction between Rhythm and Condition, and the interaction between Rhythm and ROI were also significant. The results showed significant interaction between Rhythm, Condition, and ROI factors. Post hoc pairwise analyses comparing the power between Rhythmic and Random conditions showed that in the auditory condition the effect of Rhythm was significant in the sensorimotor (*z* = 6.080, *p* = 0.002, *d* = 2.772), the temporal (*z* = 6.470, *p* = 0.002, *d* = 3.029), and occipital (*z* = 2.55, *p* = 0.013, *d* = 0.967) ROIs. In the visual condition the effect of Rhythm was also significant in the sensorimotor (*z* = 2.601, *p* = 0.011, *d* = 0.989), the temporal (*z* = 5.592, *p* = 0.002, *d* = 2.400), and the occipital ROI (*z* = 9.426, *p* = 0.002, *d* = 12.491). In the audiovisual condition the effect of Rhythm was also significant in all ROIs: sensorimotor (*z* = 6.818, *p* = 0.002, *d* = 3.310), temporal (*z* = 8.729, *p* = 0.002, *d* = 5.380), and occipital (*z* = 8.681, *p* = 0.002, *d* = 15.730) ROIs.

In addition, for the 2 and 3.6 Hz frequencies, we computed the difference in the marginal mean power between rhythmic and random sequences for each condition (audio, visual, and audiovisual) and ROI and performed pairwise comparisons to evaluate whether the rhythmic effect varied across ROI in each experimental condition (find statistical data in Table 2, see Figure 3A). The results showed that at both 2 and 3.6 Hz frequencies, in the auditory condition, the effect of rhythm was similar between the temporal and sensorimotor ROIs but was smaller in the occipital when compared with the other ROIs. In the visual condition, the highest effect of rhythm was observed in the occipital ROI compared with the sensorimotor and temporal ROIs at both 2 and 3.6 Hz frequencies. However, in line with the previous analyses suggesting that no effect of rhythm was observed in sensorimotor and temporal ROIs, no significant differences were observed between both ROIs at 2 Hz. But on the contrary, at 3.6 Hz for the visual condition, the effect of rhythm was higher in the temporal ROI compared with the sensorimotor ROI. Finally, in the audiovisual condition, at 2 Hz the effect of rhythm was similar across all sensorimotor, temporal, and occipital ROIs. In contrast, at 3.6 Hz, the results showed differences between the occipital and the sensorimotor and temporal ROIs, showing a higher activation of the occipital ROI. Further, the temporal ROI showed higher effects of rhythm compared with the sensorimotor ROI.

**TABLE 2:** Post hoc pairwise comparisons of the difference between Rhythmic and Random sequences.

Condition	Contrast between ROIs	Frequencies					
			2 Hz			3.6 Hz	
		*z*	*p*	Cohen's *d*	*z*	*p*	Cohen's *d*
Auditory	Sensorimotor vs. temporal	0.941	0.791	0.618	−0.424	0.906	−0.258
	Sensorimotor vs. occipital	5.306	**0.002**	3.396	3.114	**0.007**	1.805
	Temporal vs. occipital	4.613	**0.002**	2.778	3.509	**0.002**	2.062
Visual	Sensorimotor vs. temporal	−0.871	0.808	−0.451	−2.516	**0.036**	−1.410
	Sensorimotor vs. occipital	−5.201	**< 0.0001**	−3.475	−8.620	**< 0.0001**	−11.50
	Temporal vs. occipital	−4.505	**< 0.0001**	−3.024	−7.918	**< 0.0001**	−10.09
Audiovisual	Sensorimotor vs. temporal	0.470	0.973	0.338	−3.032	**0.009**	−2.07
	Sensorimotor vs. occipital	0.343	0.973	0.249	−7.377	**< 0.0001**	−12.41
	Temporal vs. occipital	−0.122	0.992	−0.089	−6.673	**< 0.0001**	−10.34
**Contrast between conditions**	**ROI**						
Auditory vs. visual	Sensorimotor	6.432	**< 0.0001**	4.295	3.078	**0.008**	1.783
Auditory vs. audiovisual		−0.367	0.973	−0.256	−0.882	0.677	−0.542
Visual vs. audiovisual		−6.370	**< 0.0001**	−4.551	−3.876	**0.001**	−2.325
Auditory vs. visual	Temporal	5.112	**< 0.0001**	3.226	1.056	0.584	0.629
Auditory vs. audiovisual		−0.783	0.838	−0.535	−3.451	**0.003**	−2.355
Visual vs. audiovisual		−5.357	**< 0.0001**	−3.761	−4.377	**< 0.0001**	−2.985
Auditory vs. visual	Occipital	−4.002	**0.001**	−2.576	−8.630	**< 0.0001**	−11.52
Auditory vs. audiovisual		−4.939	**< 0.0001**	−3.402	−8.178	**< 0.0001**	−14.76
Visual vs. audiovisual		−1.141	**0.694**	−0.827	−3.268	**0.005**	−3.24

*Note:* At the top, comparisons are made between ROIs for each condition. At the bottom, specific contrasts between conditions are included for the Sensorimotor, Temporal, and Occipital ROIs. *Z*‐ratios, FDR‐corrected *p*‐values, and Cohen's *d* values are reported.

We also explored differences in the effect of rhythm in each specific ROI across experimental conditions (see Table 2, Figure 3A). Specifically, for the sensorimotor ROI the results were similar at both 2 and 3.6 Hz and showed no significant differences in the effect of rhythm between the auditory and audiovisual conditions. However, there was a significant difference when comparing the visual condition with both the auditory and audiovisual conditions in the sensorimotor cortex, with higher activation observed during the auditory and audiovisual conditions (see Table 2). In the temporal ROI the effect of rhythm was higher in the auditory than in the visual condition at 2 Hz but not at 3.6 Hz. The effect of rhythm was similar between the auditory and the audiovisual condition at 2 Hz, but it was larger for the auditory at 3.6 Hz. At both frequencies the effect of rhythm was higher for the audiovisual than for the visual condition. In the occipital ROI at both 2 and 3.6 Hz the effect of rhythm was larger for the audiovisual compared with the auditory and the visual condition. At both frequencies, the effect of rhythm was larger than in the auditory.

Overall, the results from the attentional task show a higher percentage of correct answers in rhythmic sequences [auditory (77.03%), visual (85.87%), and audiovisual (91.17%)] compared with the random sequences [63.14%, 60.07%, 65.53%, respectively] (*z* = 10.67, *p* < 0.001). Interestingly, and in line with the above‐described results, participants were more accurate when being exposed to audiovisual inputs compared with the auditory alone condition (*z* = −3.09, *p* = 0.008), and when observing visual compared with auditory inputs (*z* = −4.94, *p* < 0.001). Thus, the results from the attentional task demonstrate that participants were clearly focusing attention on both rhythmic and random conditions (performance in all cases is above 60%) but being more accurate in the rhythmic ones.

## 4 Discussion

The aim of the present study was to investigate brain dynamics supporting the perception of the rhythmic sensorimotor entrainment when coupling auditory and visual inputs related to walking movement using an EEG frequency‐tagging approach. The results showed neural entrainment effects in response to the auditory and the audiovisual conditions but not in response to the visual condition; these entrainment effects were observed at a 2 Hz frequency rate. The results showed that auditory input, specifically with the footstep sounds presented in rhythmic sequences, induced an entrainment effect observed at the temporal and the sensorimotor cortex (Figure 3). In particular, the presentation of the auditory input at a 2 Hz frequency rate induced a higher entrainment peak in the sensorimotor cortex as compared with the temporal and occipital cortex (Figure 2). Moreover, exposure to the audiovisual condition at 2 Hz, which involved footsteps sounds coupled to the walking biological motion through the point‐light figure, enhanced the entrainment effect in the sensorimotor cortex (Figure 3).

The main result of the present study is that the auditory condition presented at 2 Hz, involving footstep sounds, induced higher entrainment effects within the sensorimotor cortex compared with the visual input (walking point‐light figure). It is known that the auditory system is specialized in auditory scene analysis, being an expert in segregating (segmenting) sounds that arise from different environmental auditory sources and forming internal representations of auditory objects or streams (Shamma et al. 2011). The absence of clear spatial visual cues (objects to fixate) in auditory information most probably drives this specialization in segregation and differentiation of potential auditory sources (Bregman and Campbell 1971). This is important for humans and very different from what the visual system is specialized. The visual system has been partialized into two functionally and anatomically distinct processing streams. The dorsal stream mainly processes visuospatial information, such as motion, distance, and location (the “where” or “vision for action” pathway), whereas the ventral stream focuses on object recognition (the “what” or “vision for perception” pathway) (Goodale and Milner 1992). Even though similar proposals have been made for the auditory system (dorsal and ventral what and where divisions; Rauschecker and Tian 2000; Romanski et al. 1999), the visual system is tuned to spatially localize objects in space and focalizing attention (also depending on existing spatial cues). In this regard, whereas visual scenes often present more static or slow‐moving elements, auditory scenes are more dynamic and temporally dependent, containing fast‐changing inputs and brief acoustic events (Attias and Schreiner 1997; Singh and Theunissen 2003). Hence, the auditory system emphasizes temporal integration of information, as this allows the system to decode the sources and achieve segregation (Shamma et al. 2011).

Although the integration of auditory input in time, mediated by temporal coherence computations, can occur with minimal cognitive control, attentive listening is crucial for integrating coherent attributes across the different streams (dorsal and ventral routes of auditory processing). In this regard, some studies have shown that when one attends to incoherent sound inputs of alternating sounds, one initially perceives a unified percept (Kramer and Bregman 1992), and incoherence is often ignored prior to the onset of attention. Therefore, attention is a crucial aspect in both auditory and visual scene analyses for perceptual sensory input integration over time. In line with these findings, our study showed higher integration of both auditory and visual inputs in rhythmic sequences compared with random sequences (see Figures S1 and S2). Further, rhythmic sequences of the auditory input (footstep sounds) induced higher entrainment with the sensorimotor cortex compared with rhythmic sequences of the visual input (walking point‐light figure). This difference might be related to the differential role of attention in auditory and visual scene analysis. Selective visual attention has a fundamental role in visual search, as we are unable to fully process everything in the scene simultaneously (Keshvari and Rosenholtz 2016). In this regard, it has been shown that two elements that do not guide attention for feature search in a visual scene are biological motion and visual rhythms (Wolfe and Horowitz 2017). Then, if the visual input (point‐light figure) did not trigger exogenous attention, this may explain why it did not induce a similar entrainment effect with the sensorimotor cortex as that observed for the other conditions in our study.

Nevertheless, the results show that in the audiovisual condition, there is an enhancement of the activation peak in the sensorimotor cortex at 2 Hz (see Figure 3). This is in line with previous investigations where an enhanced amplitude was observed, driven by synchronous auditory and visual input delivered at temporal rates of ~2 Hz (Nozaradan et al. 2012). These cited results were interpreted in line with the notion of increased attentional gain due to audiovisual synchrony. In the present study, as in previous investigations (Keitel and Müller 2016), the visual input presented sustained amplitude gain effects in the sensorimotor and occipital cortex when presented synchronously with the auditory input. This result supports the growing body of literature on the interplay of attention and multisensory integration (Talsma et al. 2010). Indeed, the results from the attentional task, showing a higher percentage of correct answers in rhythmic sequences, are in convergence with the Dynamic Attending Theory defined by Jones and Boltz (1989), that defines synchronous interactions as a dynamic activity that involves the interaction of an attender's internal (cortical) rhythms with the external stimulus rhythms (van Wassenhove and Herbst 2020). Thus, any perceptual stimuli in phase with a peak of attention are more expected (Ellis and Jones 2010), and are thus better processed (see also (Bolger et al. 2014)). Moreover, in line with our results showing higher attention on audiovisual inputs, previous studies have shown cross‐modal benefits when coupling auditory meter rhythms with visual inputs (Escoffier et al. 2010). The authors reported that observers were faster at discriminating between upright and upside‐down pictures (faces or buildings) when the visual stimulus was synchronized on beat (in synchrony with a strong metric position of a background auditory rhythm), as compared with when it was displayed off‐beat (out of synchrony with the implied musical pulse) (Escoffier et al. 2010).

Finally, another important finding of this study is that we observed a higher neural entrainment effect (the coupling of internal oscillations to rhythmic external events; Obleser and Kayser 2019), in the sensorimotor cortex. In both auditory and audiovisual conditions, this effect was obtained when the sensory inputs were presented at 2 Hz. Interestingly, related to human locomotion, the 2 Hz frequency has also been shown to be related to the spontaneous intrinsic tempo of a spinal central pattern generator (CPG). CPGs have been established as the basis for locomotor rhythmicity in invertebrates, primitive fish, and cats (Grillner 1985, 1986; Grillner and Wallen 1985), although their existence in humans can only be inferred from indirect evidence (Dietz 2003; Marder 2001). Related to music, a study of western music from the latter half of the 20th century demonstrated a clear preference for musical tempi around 120 BPM (2 Hz) (Moelants 2002), and other studies suggest that vertical motion at 2 Hz may indeed be perceived as pleasurable (Todd and Cody 2000). According to this, previous investigations in the domain of beat perception have also found sensorimotor coupling with auditory areas at a 2.4 Hz frequency rate (Nozaradan et al. 2015). In that study, the authors used a finger‐tapping synchronization task to explore the neural dynamics supporting sensorimotor synchronization to the beat, that is, the performance of overt movements paced on the beat using steady‐state‐evoked potentials (SS‐EPs). The authors found that when subjects tapped to the beat, the EEG was characterized by a 2.4 Hz SS‐EP compatible with beat‐related entrainment, involving a dynamic coupling of sensory and movement‐related neuronal entrainments (Nozaradan et al. 2015). In addition, previous investigations using auditory stimuli have found an entrainment of neuronal populations at the frequency of the beat and at the subharmonics corresponding to the metric interpretation of this beat, presented at 2.4 Hz periodicity (Nozaradan et al. 2011).

Regarding visual input from biological movement perception, it has been proposed that this perception could be mediated by a top‐down mechanism that produces a percept of biological motion even in the absence of any retinal motion (Orgs and Haggard 2011). These mechanisms typically make use of both shape cues (i.e., the body postures composing the movement) and motion cues (i.e., the motion patterns of the different body parts; Giese and Poggio 2003); but can also detect biological movements from motion cues alone (Giese and Poggio 2003). To study these mechanisms, research often uses point‐light figures, a type of stimuli that represents human movements as a set of moving dots placed on the major joints (Blake and Shiffrar 2007). Following this approach, a recent study used a ‘point‐light walker’ to define the brain responses associated with biological motion perception using an EEG frequency‐tagging approach (Cracco et al. 2022). In detail, the point‐light walker was moving at a pace of 2.4 Hz and EEG was used to measure the brain response coupled to that pace (Cracco et al. 2022). The results revealed a reliable response at the walking frequency (2.4 Hz) in the occipital cortex (Cracco et al. 2022), in line with the present study's findings. However, in our study, we observed a larger peak at 3.6 Hz in the occipital cortex during the visual and audiovisual conditions (see Figure 2) compared with the auditory condition. According to this result, it is known that audiovisual synchrony can act as a strong attractor for spatial attention. For example, presenting visual stimuli (Gabor patches) that differed in color and orientation, together with an auditory cue to selectively attend to one of the two patches presented at distinct rates at 3.14 and 3.63 Hz, selectively enhanced cortical processing in the parieto‐occipital cortex (Keitel and Müller 2016). Indeed, some studies on multisensory interactions during synchronous presentation of auditory and visual cues have established a speed limit of < 4 Hz for the perception of matching features for audiovisual synchrony (Fujisaki and Nishida 2008). Therefore, we can postulate that the peak at 3.6 Hz in the auditory and audiovisual conditions is related to the matching of salient temporal features selected from each sensory modality (point‐light figure and footstep sounds) by top‐down attention mechanisms to a specific spatial position of the audiovisual sensory inputs (Fujisaki and Nishida 2008). Other studies have also shown that the temporal resolution of synchrony perception for audiovisual stimuli and the neural correlations of perceptual synchrony across different multisensory areas responding to audiovisual stimuli presented in a repetitive pulse can be processed only if the stimulus temporal frequency is below 4 Hz (Fujisaki and Nishida 2005, 2009). However, a study aimed at investigating the stimulus presentation rate at which the human brain can discriminate between different types of familiar visual category (faces) suggests that stimulation rates between 3.5/4 and 6 Hz may elicit the largest differences between repeated and novel individual faces in the right occipito‐temporal cortex (Alonso‐Prieto et al. 2013). Hence, while we may speculate that the peak observed at 3.6 Hz, especially in the occipital and the temporal brain areas, could be related to the processing or recognition of familiar visual inputs, the peak at 2 Hz in the sensorimotor and temporal brain areas is more relevant to our study, as it is more directly related to the perception of rhythmic stimulation associated with the walking movement.

The results from our study show auditory‐driven coupling in the sensorimotor and temporal ROIs predominates at 1 Hz, this result is in line with previous investigations in which auditory beats at 1.2 Hz induced motor responses (Colling et al. 2017; Nozaradan et al. 2015). However, interestingly, audiovisual binding and widespread activation across all ROIs emerged only at 2 Hz. This result is in line with previous results supporting the effectiveness of using audiovisual (or multimodal) stimulation to enhance sensorimotor entrainment compared with using auditory or visual inputs alone (Drijvers et al. 2021). Specifically, Drijvers and colleagues used rapid invisible frequency tagging to generate steady‐state evoked fields and observed the integration of audiovisual information in a semantic context task. The study showed a clear spectral peak at the intermodulation frequency of the auditory and visually tagged signals, specifically when lower‐order integration was easiest because signal quality was optimal. Such enhanced power at the intermodulation frequency showed the ease of lower‐order audiovisual integration in a speech (audio)‐gesture (visual) task in sensory cortices. This finding is in line with our results showing that merging footstep sound (audio) and walking point‐light figure (visual) inputs presented at 2 Hz induced higher integration in sensorimotor cortices. However, other studies did not find such effects in the SSEPs presenting temporally congruent audiovisual inputs compared with unimodal and incongruent bimodal conditions (Lapenta et al. 2023). In the study by Lapenta and co‐authors, the authors argue that the absence of clear landmarks on the auditory loudness modulation in their study may influence auditory‐motor coupling, as well as the instruction to focus on the visual stimuli information disregarding the auditory one, which could have contributed to the null findings among audiovisual conditions. In our study, even though the participants in the audiovisual task had to pay attention to the central red point (counting the number of white color changes) we observed greater entrainment effects especially at 2 Hz. The results from the present study can be crucial for both unimodal and multimodal entrainment effects in the sensorimotor cortex. Hence, in this paradigm, unimodal entrainment appears more prominent at 1 Hz, whereas audiovisual integration is more robust at 2 Hz, depending on the stimulus properties and underlying neural organization. Such results highlight the existence of a frequency‐specific window for sensorimotor integration and multimodal binding. Indeed, similar frequency dependent effects might be expected for other ecological audiovisual actions beyond walking, including tool use or speech–gesture combinations, under appropriate frequency‐tagging schemes. These results may pave the way to other types of audiovisual stimulation applications, by which choosing proper stimulation frequencies tuned to natural body action rhythms could optimize entrainment based sensorimotor training or rehabilitation protocols, by improving the effectiveness of multisensory integration interventions.

## 5 Limitations of the Study

The results from this study have some limitations as the participants were sitting without moving while observing or hearing the sensory inputs. To further explore the effects of sensory inputs on rhythmic sensorimotor entrainment, further studies adding synchronous body movements to the presentation of both auditory and visual inputs are needed. Moreover, another potential limitation of the study is on the attentional tasks used in the audiovisual condition; we only included the central point color change as the attentional measure (like in the visual alone condition), omitting the pitch changes. In this sense, this manipulation might have affected the visual more than auditory domains in terms of attentional focus. Further studies might be needed to disentangle if adding more complex attentional tasks involving auditory and visual changes in the audiovisual condition might affect EEG entrainment differently. Another limitation of the study would be that we did not use a non‐biological control condition; even though we have followed the methodology used in previous studies (Cracco et al. 2022; Formica et al. 2024; Warlop et al. 2024), a non‐biological control condition would allow us to test whether the observed effects depend on the biological motion content.

In conclusion, this study provides potential evidence of the impact of auditory, visual or audiovisual inputs delivered in rhythmic walking sequences on the sensorimotor cortex. The results show evidence about how auditory inputs can synchronize auditory brain areas (temporal cortex) with the sensorimotor cortex. Specifically, footsteps sound presented at 2 Hz induced a peak amplitude for coupling with the sensorimotor cortex. Further, when footstep sounds were combined with a walking point‐light figure (presented at the same frequency rate), it induced higher coupling with the sensorimotor cortex. However, we cannot attribute any sensory modality specificity to the results obtained in this study, but rhythmical entrainment effects with brain oscillatory activity. Indeed, these results are in line with previous investigations showing highly tuned 2 Hz vertical acceleration of the head and body during active locomotion that could be mediated by a central ‘resonant frequency’ of human movement. However, further research is needed to understand the role of auditory inputs in tuning sensorimotor cortex coupling compared with visual ones. The results from this study may be relevant for designing sensory learning and multisensory integration trainings for motor rehabilitation, based on the neurobiology of rhythmic motor entrainment (Molinari et al. 2003), particularly for the rehabilitation of rhythmic movements such as walking movement.

## Author Contributions

**Marta Matamala‐Gomez:** conceptualization, investigation, funding acquisition, writing – original draft, methodology, validation, visualization, writing – review and editing, data curation, software. **Adrià Vilà‐Balló:** writing – original draft, methodology, validation, visualization, writing – review and editing, formal analysis, data curation, project administration. **David Cucurell:** data curation, formal analysis, software. **Ana Tajadura‐Jiménez:** conceptualization, funding acquisition, writing – review and editing, supervision, validation, project administration. **Antoni Rodriguez‐Fornells:** conceptualization, funding acquisition, methodology, validation, supervision, project administration.

## Funding

M.M.‐G. receives funding from the European Union through HORIZON‐MSCA‐2022‐PF‐01, Num: 101110198. The cognition and Brain Plasticity Group is funded by Generalitat de Catalunya 2021SGR01099 led by A.R.‐F. ARF has been supported by the FIAS fellowship Program, co‐funded by the European Commission, Marie‐Skłodowska‐Curie Actions ‐ COFUND Program, Grant n°945408. A.T.‐J. receives funding from the European Research Council (ERC) under the European Union's Horizon 2020 research and innovation program (Grant Agreement No. 101002711; project BODYinTRANSIT).

## Conflicts of Interest

The authors declare no conflicts of interest.

## Supporting information


**Data S1:** psyp70225‐sup‐0001‐Supinfo.docx.

## Data Availability

The data that support the findings of this study are openly available in OSF at https://osf.io/8sa9t/?view_only=b5d74f54b51f4793b8ab915540b1ab54, reference number DOI 10.17605/OSF.IO/8SA9T.
