# Supplementary material for: Sensorimotor Frequency Tagging Is Enhanced by Auditory and Audiovisual but Not Visual, Inputs During a Body‐Walking Task
Source: Psychophysiology. 2026 Jan 28;63(2):e70225. doi: 10.1111/psyp.70225 (PMC12851424; doi:10.1111/psyp.70225)
Supplement: Supplementary file 1 — Data S1: psyp70225‐sup‐0001‐Supinfo.docx. [file PSYP-63-e70225-s001.docx]

**Supplementary Materials**

**Supplementary method**

Spectral power was computed using the FieldTrip toolbox in MATLAB, employing the mtmfft method [multi-taper method (MTM) with a discrete prolate spheroidal sequence (DPSS) taper and a fast Fourier transform (FFT)], using a Hanning window as the taper. For each frequency bin *f*, a local baseline was defined using 10 bins on either side, excluding the two bins directly adjacent to f to avoid contamination from spectral leakage. Given the frequency resolution of 0.05 Hz, this meant excluding bins at ±0.05 Hz and using bins from ±0.10 Hz to ±0.55 Hz relative to *f*. To reduce the influence of outliers, the minimum and maximum values within the selected baseline bins were excluded before computing the baseline mean (μ*_f_*) and standard deviation (σ*_f_*). The z-score (Z*_f_*) at frequency f was then calculated as:

Z*_f_* = (P*_f_* − μ*_f_*) / σ*_f_*

where P*_f_* is the spectral power at bin *f*, μ*_f_* is the trimmed mean of the baseline bins, and σ*_f_* is the trimmed standard deviation. This procedure was repeated for all frequency bins and electrodes, ensuring consistent standardization across the spectrum and participants.

We statistically analysed the z-score data using a linear mixed-effects model (LMM) that included Rhythm (Rhythmic, Random), Condition (Auditory, Visual, Audiovisual), ROI (Sensorimotor, Temporal, Occipital), and Frequency (1 Hz, 2 Hz, 3.6 Hz) as within-subject factors, along with all possible interactions. Participant was included as a random intercept to account for individual differences. For the analyses, the three ROIs comprised the following electrodes: Temporal (right: FT9, FT7, T7, TP7; left: FT10, FT8, T8, TP8), Sensorimotor (right: F5, F3, F1, FC5, FC3, FC1, C5, C3, C1; left: F2, F4, F6, FC2, FC4, FC6, C2, C4, C6), and Occipital (PO3, POz, PO4, O1, Oz, O2). The model was fitted using a Gamma distribution with an inverse link function, which is appropriate for the positively skewed continuous response variable (Power). Optimization was performed using the “bobyqa” algorithm.

**Supplementary Results**

To directly assess whether the effect of rhythmic differed depending on the spectral frequency of neural activity, we conducted a supplementary linear mixed-effects model (LMM) including Frequency (1 Hz, 2 Hz, 3.6 Hz) as an additional within-subject factor alongside Rhythm, Condition, and ROI. The full factorial model included all predictors and their interactions, and the results of this analysis are presented in Supplementary Table S1.

Significant main effects of Rhythm, Condition, ROI, and Frequency were observed. Specifically, z-score values were generally higher for Rhythmic compared to Random sequences, confirming an overall enhancement in power under rhythmic stimulation. Moreover, differences across Conditions showed that the neural response changed depending on the modality of stimulation, while effects across ROIs revealed that some cortical regions exhibited stronger responses than others. Finally, the main effect of Frequency reflected systematic differences in power across the 1 Hz, 2 Hz, and 3.6 Hz bands. Some two-way and three-way interactions involving Rhythm also reached significance (e.g., Rhythm × Frequency; Rhythm × Condition × ROI; Rhythm × ROI × Frequency), whereas others did not (e.g., Rhythm × Condition; Condition × ROI × Frequency), suggesting that the modulation induced by rhythmic stimulation varied to some extent across conditions, regions, and frequencies. However, these effects should be interpreted within the broader context of the full model, especially considering the significant four-way interaction (Rhythm × Condition × ROI × Frequency). This interaction indicates that the effect of Rhythm on spectral power was not uniform but depended jointly on stimulation modality, cortical region, and frequency band. In other words, rhythmic stimulation did not produce a global increase in power across all frequencies and ROIs; rather, its impact was frequency-specific and spatially differentiated. Accordingly, this justifies analysing each frequency separately using reduced models that include Rhythm, Condition, and ROI as predictors for each frequency band, as presented in the main text.

**Table S1.** Chi-square values and p-values (Pr(>Chisq)) obtained from the full model including Rhythm (Rhythmic, Random), Condition (Auditory, Visual, Audiovisual), ROI (Sensorimotor, Temporal, Occipital), and Frequency (1 Hz, 2 Hz, 3.6 Hz) as within-subject factors. The table reports main effects and all interactions. Degrees of freedom (df) and Absolute values of Cohen’s d are reported.

| **Effect** | **Chisq** | **Df** | **Pr(>Chisq)** | **Cohen’s d** |
| --- | --- | --- | --- | --- |
| Rhythm | 65.2841 | 1 | 6.484e-16 *** | 0.484 |
| Condition | 33.9805 | 2 | 4.180e-08 *** | 0.349 |
| ROI | 6.3012 | 2 | 0.042827 * | 0.15 |
| Frequency | 28.3974 | 2 | 6.817e-07 *** | 0.319 |
| Rhythm × Condition | 3.6805 | 2 | 0.158781 | 0.115 |
| Rhythm × ROI | 5.4718 | 2 | 0.064834 . | 0.14 |
| Condition × ROI | 35.2782 | 4 | 4.072e-07 *** | 0.356 |
| Rhythm × Frequency | 31.912 | 2 | 1.176e-07 *** | 0.339 |
| Condition × Frequency | 8.251 | 4 | 0.082805 . | 0.172 |
| ROI × Frequency | 24.9832 | 4 | 5.070e-05 *** | 0.3 |
| Rhythm × Condition × ROI | 24.6721 | 4 | 5.855e-05 *** | 0.298 |
| Rhythm × Condition × Frequency | 14.424 | 4 | 0.006058 ** | 0.228 |
| Rhythm × ROI × Frequency | 37.7729 | 4 | 1.248e-07 *** | 0.368 |
| Condition × ROI × Frequency | 11.8316 | 8 | 0.158879 | 0.206 |
| Rhythm × Condition × ROI × Frequency | 17.3032 | 8 | 0.027102 * | 0.249 |

Note: Significance codes: *** p < 0.001, ** p < 0.01, * p < 0.05

**Supplementary data figures**

In this supplementary material, we present the normalized power spectra for the Rhythmic and Random sequences (Figures 1-1 and 2-1), separated by ROI and condition, including visual marking of the target peak frequencies used for the subsequent analysis. Additionally, Figure 3-1 shows the normalized spectral power at each stimulation frequency, separately for Rhythmic and Random sequences, allowing for visualization across ROIs and conditions.


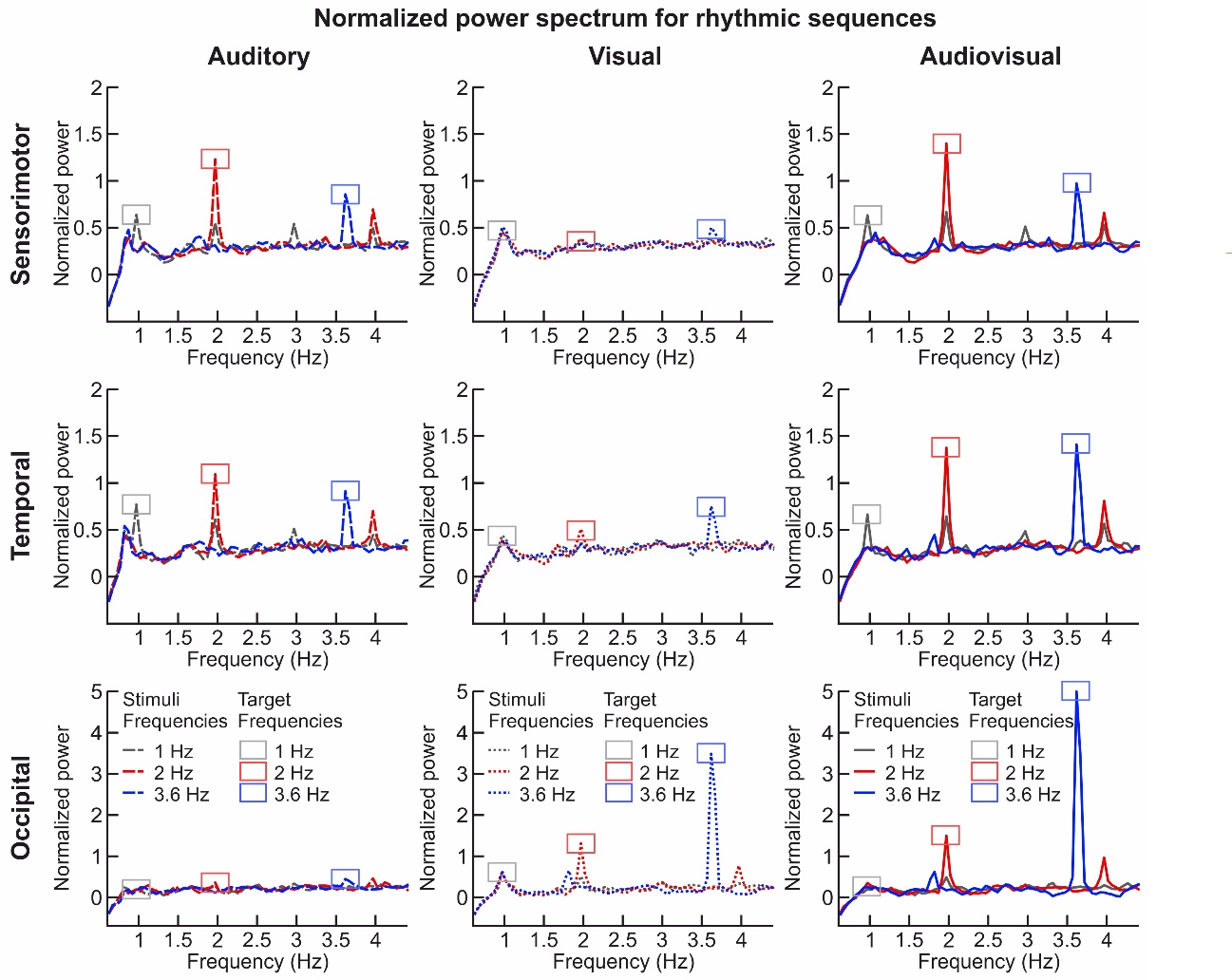


**Figure 1-1.** The figure illustrates the normalized average power spectrum of Rhythmic sequences, separated for sensorimotor (top), temporal (middle), and occipital (bottom) regions of interest (ROIs), and for auditory (left, dashed lines), visual (middle, dotted lines), and audiovisual (right, solid lines) conditions. Stimulation frequencies are represented as follows: grey for 1 Hz, red for 2 Hz, and blue for 3.6 Hz. Rectangles indicate the target peak frequencies that will be included in Figure 3 - 1 (grey for 1 Hz, red for 2 Hz, and blue for 3.6 Hz). Spectral power was normalized by the mean power across frequencies, and baseline corrected by first subtracting and then dividing by the mean of the adjacent bins (two on each side, spaced two bins from the target one).


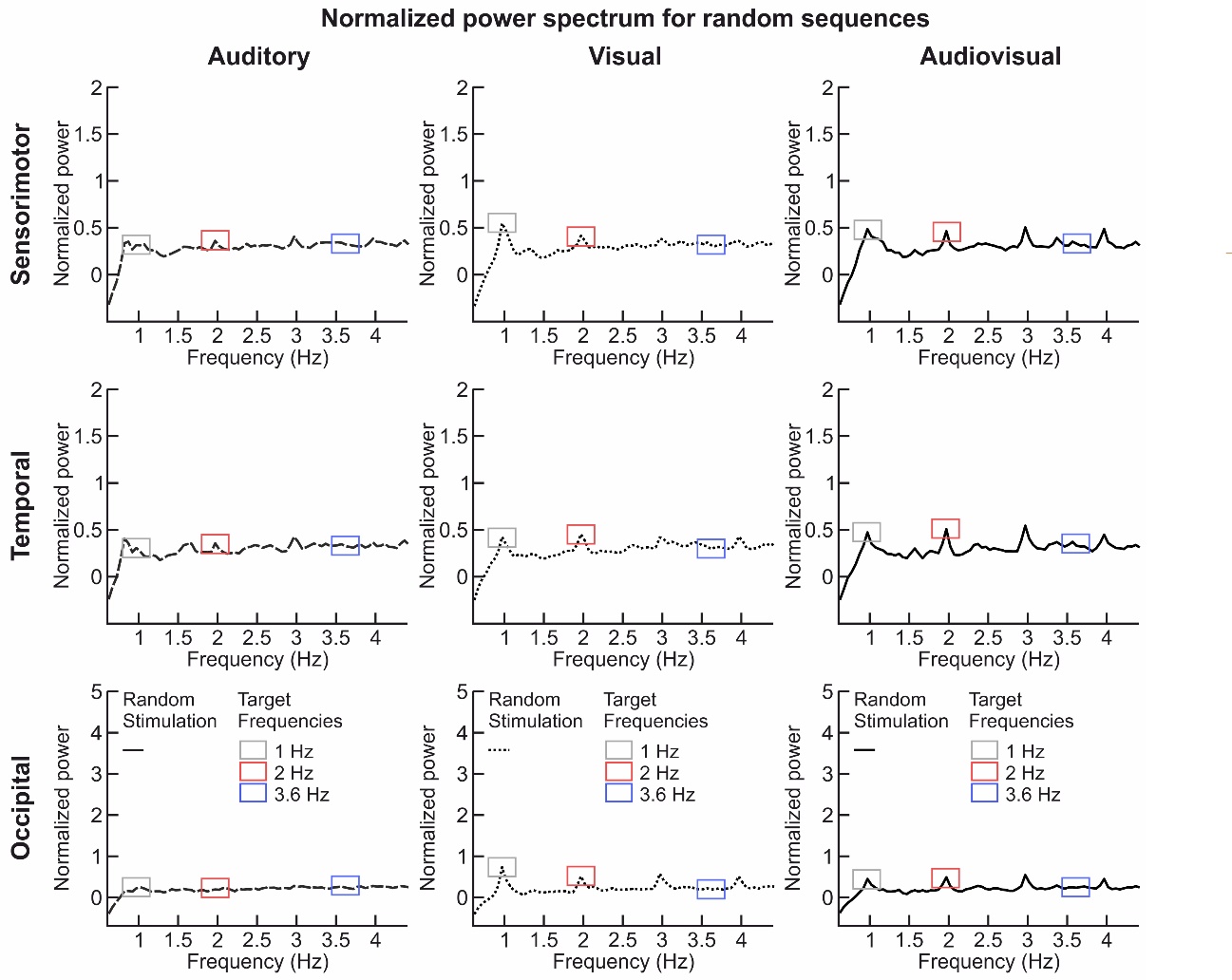


**Figure 2-1.** The figure illustrates the normalized average power spectrum of Random sequences, separated for sensorimotor (top), temporal (middle), and occipital (bottom) regions of interest (ROIs), and for auditory (left, dashed lines), visual (middle, dotted lines), and audiovisual (right, solid lines) conditions. All power spectrum lines are represented in black. Rectangles indicate the target peak frequencies that will be included in Figure 3 - 1 (grey for 1 Hz, red for 2 Hz, and blue for 3.6 Hz). Spectral power was normalized by the mean power across frequencies, and baseline corrected by first subtracting and then dividing by the mean of the adjacent bins (two on each side, spaced two bins from the target one).


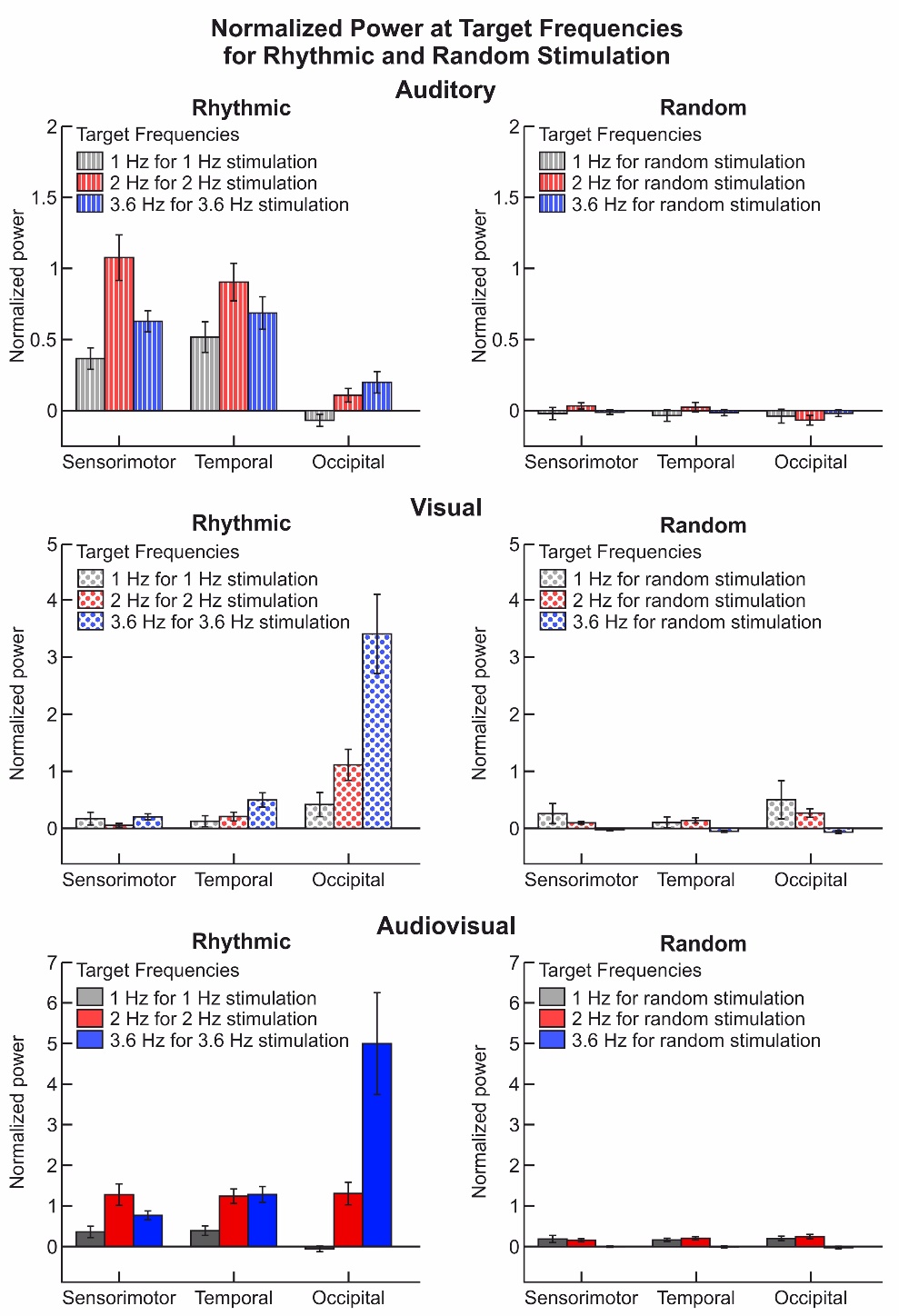


Figure 3-1. The figure illustrates the normalized average power spectrum for each target frequency (1 Hz, 2 Hz, or 3.6 Hz) at each corresponding stimulation frequency (1 Hz, 2 Hz, or 3.6 Hz), shown separately for rhythmic (left) and random (right) sequences. Bar plots for sensorimotor, temporal, and occipital regions of interest (ROIs), as well as for auditory (top, represented with dashed bars), visual (middle, represented with dotted bars), and audiovisual (bottom, represented with solid bars) conditions, are shown. For rhythmic sequences, the spectral power at 1 Hz is displayed only for the stimulation frequency of 1 Hz (colored grey), at 2 Hz only for the stimulation frequency of 2 Hz (colored red), and at 3.6 Hz only for the stimulation frequency of 3.6 Hz (colored blue). For random sequences, we selected the same target peaks and used the same colors, even though there is no specific stimulation frequency. Error bars represent the standard errors of the mean (SEMs). Spectral power was normalized by the mean power across frequencies, and baseline corrected by first subtracting and then dividing by the mean of the adjacent bins (two on each side, spaced two bins from the target one).
